# GSC-YOLO: A Pedestrian Detection Method for Low-Light Security Surveillance Scenarios

**DOI:** 10.3390/s26102987

**Published:** 2026-05-09

**Authors:** Wei Qing, Fan Li, Shuang Li, Pengfei Yin

**Affiliations:** 1 College of Computer Science and Engineering, Jishou University, Jishou 416000, China; 2023404585@stu.jsu.edu.cn (W.Q.); lishuang@stu.jsu.edu.cn (S.L.); 2 College of Communication and Electronic Engineering, Jishou University, Jishou 416000, China; 2023403728@stu.jsu.edu.cn

**Keywords:** low-light pedestrian detection, GSC-YOLO, nighttime visual perception

## Abstract

Pedestrian detection in nighttime security surveillance and other low-light visual sensing tasks is an important foundation for intelligent perception in complex environments. Under low-light conditions, visible-light images often suffer from missing texture details, intensified noise, and reduced contrast, which can easily lead to insufficient target representation, unstable cross-scale feature fusion, and an increased risk of missed detections. Although multimodal schemes, such as RGB–infrared approaches, can improve detection performance by exploiting modal complementarity, they involve relatively high hardware costs, cross-modal calibration complexity, and system integration overhead, which impose deployment limitations in lightweight or cost-sensitive scenarios. Therefore, developing an efficient pedestrian detection method for low-light monocular RGB scenarios is of clear practical value. This study focuses on low-light monocular RGB pedestrian detection and proposes an application-oriented structurally optimized model, termed GSC-YOLO, built upon YOLOv13. First, GhostNetV3 is introduced as the backbone to enhance multi-scale feature representation under weak-texture conditions. Second, a Semantic–Spatial Alignment (SSA) module is designed to improve information compensation and suppress noise during the feature fusion stage. Finally, C2f_Faster is incorporated into the high-level semantic branch to optimize information flow and reduce redundant computation. On the RGB subsets of the two public datasets, LLVIP and KAIST, GSC-YOLO achieves mAP@0.5:0.95 values of 57.70% and 66.61%, respectively, and Recall values of 89.93% and 90.49%, respectively, consistently outperforming the YOLOv13 baseline. The results demonstrate that, under the experimental settings adopted in this study, the proposed method effectively improves pedestrian perception performance in low-light RGB scenes while maintaining favorable real-time inference capability, and may provide a useful reference for front-end vision sensing research in low-altitude intelligent networks.

## 1. Introduction

Robust nighttime pedestrian detection is a fundamental capability for public safety monitoring, nighttime security inspection and patrol, and intelligent perception in complex environments [1]. With the development of low-altitude intelligent sensing and UAV-based inspection, low-altitude platforms have shown increasing potential in related nighttime applications [2]. However, the present study focuses on low-light monocular RGB pedestrian detection itself, and the low-altitude scenario is considered only as a potential application context rather than an experimentally validated deployment setting. Low-light visible-light images commonly suffer from reduced signal-to-noise ratio, missing texture details, and contrast degradation [3,4], which significantly undermine the reliability of target perception. Therefore, investigating robust pedestrian detection methods for low-light monocular RGB imagery is of clear practical value and may also provide a useful reference for future research on low-altitude nighttime visual perception.

To enhance nighttime visual perception, RGB–infrared fusion methods have attracted widespread attention because of their modal complementarity [5,6]. However, such methods usually rely on dual-camera hardware and complex cross-modal calibration and registration, which increase system cost and deployment complexity and also limit their application on lightweight inspection platforms [7]. In addition, some multimodal studies place greater emphasis on image fusion quality, while insufficient attention has been paid to the optimization of downstream detection performance, real-time capability, and deployment efficiency [8]. Therefore, developing a monocular RGB pedestrian detection method that balances detection accuracy, recall, and computational cost without introducing additional sensors is of clear practical significance.

From the perspective of intelligent perception systems, nighttime pedestrian detection is not only a low-light object detection problem, but also an important component of scene perception. Stable pedestrian localization under complex illumination conditions directly affects risk assessment, interaction understanding, and motion planning. Previous studies [9] have shown that perception quality and scene understanding capability are strongly coupled in intelligent transportation systems, and have also highlighted the increasingly important role of intelligent perception and data-driven semantic understanding in pedestrian traffic analysis and decision support [10]. In low-altitude intelligent networks, environmental perception likewise serves as the foundation linking target understanding, cooperative control, and task execution. Although this study does not involve control strategies, communication coordination mechanisms, or onboard UAV validation, it can still be regarded as a foundational methodological study for front-end environmental perception in low-altitude intelligent networks.

Among existing nighttime vision techniques, image-enhancement-based preprocessing strategies (e.g., brightness adjustment, gamma correction, and Retinex) can improve visual appearance; however, they often fail to recover missing details and may even amplify noise or introduce artifacts, thereby interfering with subsequent detection [11,12]. From an architectural perspective, mainstream multi-scale feature fusion networks such as FPN [13] and PAN [14] typically integrate cross-level information via simple concatenation (Concat) or linear weighting. Under low-light and low-SNR conditions, such coarse-grained fusion tends to propagate low-level noise into high-level semantic branches, weakening the model’s ability to recognize small, weak-saliency, and occluded targets. Meanwhile, attention mechanisms such as SE [15], CA [16], and CBAM [17] can improve feature selectivity. Nevertheless, they are often used as standalone modules with insufficient collaborative modeling between semantic cues and spatial details, leaving room for improvement in noise suppression and weak-target enhancement under complex nighttime backgrounds.

The YOLO family of one-stage detectors has shown strong potential for practical object detection due to its favorable balance between accuracy and real-time performance. However, its detection performance can still be improved in nighttime scenes involving low illumination, occlusion, and complex backgrounds. In particular, lightweight variants often reduce network depth and width to accelerate inference, which constrains representational capacity and makes the detector more prone to missed detections and false alarms, making it difficult to simultaneously achieve high precision and recall. Therefore, achieving a reasonable balance among detection accuracy, model complexity, and deployment efficiency remains a key challenge for nighttime pedestrian detection.

To address issues such as insufficient semantic support for weak targets, noise propagation during cross-layer fusion, and redundancy in high-level semantic representations, this study proposes an application-oriented structurally optimized model, termed GSC-YOLO, based on the YOLOv13 architecture [18], and validates its effectiveness on the RGB subsets of public datasets, including LLVIP [19] and KAIST [20]. It should be emphasized that this study does not simply combine existing modules; rather, it performs targeted collaborative optimization of backbone representation, cross-layer fusion, and high-level semantic aggregation around the typical failure mechanisms of low-light monocular RGB pedestrian detection. The main contributions are summarized as follows:

(1) We propose the Semantic–Spatial Alignment (SSA) module. This module is designed as a sequential “compensate-then-refine” mechanism. By performing semantic–detail interaction before channel–spatial recalibration, SSA effectively improves the effectiveness and stability of fused features in low-light scenes.
(2) We construct the GSC-YOLO synergistic optimization framework. Through the strategic integration of GhostNetV3 [21], SSA, and C2f_Faster, the framework improves multi-scale representation, cross-layer fusion quality, and high-level semantic information flow efficiency, respectively.
(3) Systematic experiments were conducted on the RGB subsets of the LLVIP and KAIST datasets. The results demonstrate that the proposed method achieves a favorable balance among accuracy, recall, and efficiency in low-light visible pedestrian detection, offering a methodological reference for front-end visual sensing research in nighttime security surveillance and low-altitude intelligent networks.

## 2. Related Work

This section briefly reviews recent advances in one-stage object detection for nighttime and low-light scenes, and discusses the applicability of lightweight backbones, multi-scale fusion strategies, and attention mechanisms in complex environments. Nighttime monocular RGB pedestrian detection faces challenges such as weak texture, severe noise, and unstable cross-scale information propagation. Therefore, improving feature representation and fusion quality remains a key issue in this task.

### 2.1. Evolution of Object Detection Algorithms

In recent years, object detection has continuously evolved from two-stage frameworks to one-stage frameworks. One-stage detectors represented by YOLO (You Only Look Once) [22,23,24] directly perform category prediction and bounding-box regression on feature maps, substantially improving inference efficiency while maintaining competitive accuracy. As a result, they have become a mainstream solution for real-time object detection. From YOLOv1 [25] to recent iterations such as YOLOv10 [26], YOLOv11 [27], and YOLOv12 [28], the YOLO series [29] has continuously improved the accuracy–speed trade-off by introducing CSPNet, ELAN-style designs, anchor-free mechanisms, and more efficient label assignment strategies. However, most existing studies focus on detection under normal illumination, while research on maintaining feature diversity, suppressing noise interference, and strengthening weak-target representations in extremely low-light conditions remains relatively limited [30,31,32]. Therefore, building one-stage detection frameworks that are both robust and real-time for nighttime scenarios remains of significant research value.

### 2.2. Backbone Network Design

The backbone determines the quality of fundamental feature extraction and is a key component influencing overall detector performance. To meet edge-deployment requirements, lightweight networks such as MobileNet [33] and ShuffleNet [34] reduce computation via depthwise separable convolutions, channel shuffling, and grouped computation, improving deployability on mobile and embedded platforms. Meanwhile, GhostNet generates additional features using inexpensive operations to obtain richer representations with lower overhead [35]. However, in low-light nighttime scenarios, low computational complexity does not necessarily imply high detection robustness. Due to weak texture, low contrast, and strong noise, feature responses tend to be less stable, and small or weak-saliency targets are more easily overwhelmed by complex backgrounds, reducing the separability and usability of backbone features. In addition, practical efficiency depends not only on FLOPs or parameter counts but also on operator types, memory-access patterns, and hardware parallelism friendliness. Consequently, theoretical complexity and real-world inference speed do not always exhibit a strict linear relationship [36,37]. Therefore, backbone design for nighttime detection should emphasize a holistic balance among representational capacity, runtime efficiency, and deployment friendliness.

### 2.3. Multi-Scale Feature Fusion

Multi-scale feature fusion is an essential component of modern detectors for improving scale generalization and adaptability to complex scenes. FPN and PAN enhance high-level semantic propagation and low-level localization cues through top-down and bottom-up pathways, respectively, enabling detectors to better satisfy recognition and localization requirements across object scales. However, under low-light nighttime conditions, such coarse-grained fusion still exhibits notable limitations. Features from different levels differ substantially in semantic depth, spatial resolution, and noise distribution. If cross-level fusion relies solely on simple concatenation or linear combination, channel redundancy increases and low-level noise is propagated into high-level semantic branches, undermining stable detection of small, weak, and occluded targets. In nighttime scenes with uneven illumination, complex backgrounds, or strong local reflections, such simple fusion may further cause dispersed target responses and insufficient saliency, thereby increasing the risk of missed detections [38,39]. Therefore, introducing more effective cross-level interaction mechanisms and information-selection strategies during multi-scale fusion has become an important research direction for improving nighttime detection performance.

### 2.4. Attention Mechanisms

Attention mechanisms adaptively recalibrate feature responses along the channel dimension, the spatial dimension, or both, thereby enhancing discriminability, encouraging the model to focus on target-related regions, and suppressing irrelevant background interference [40,41,42]. In recent years, representative modules such as SE, CA, and CBAM have been widely applied to image classification, object detection, and semantic segmentation. Nevertheless, existing attention mechanisms still have several limitations in nighttime detection applications. First, attention modules introduce additional computation, and frequent stacking across multiple levels may adversely affect real-time inference. Second, many attention methods recalibrate single-level features without explicitly modeling cross-scale semantic interactions, making it difficult to fundamentally alleviate the tension between insufficient deep semantics and strong shallow noise under weak-texture nighttime conditions. Finally, if upstream fusion lacks effective filtering, attention may even amplify background noise or induce unstable responses. Therefore, co-designing attention with cross-scale information-flow organization while controlling the additional computational burden is a more practically valuable direction for real-time nighttime detection.

In summary, evaluating nighttime monocular RGB detection should not be limited to a single accuracy metric or subjective visual quality; it should also emphasize stability under complex backgrounds, small targets, and cross-scene generalization, and report deployable accuracy–efficiency trade-offs using efficiency metrics. Building on these insights, we propose GSC-YOLO. Within the YOLOv13 [18] framework, we design an SSA module to enable cross-scale semantic interaction and adaptive noise suppression, adopt GhostNetV3 to strengthen multi-scale backbone representations, and employ C2f_Faster to enhance high-level semantic aggregation and information-flow efficiency. The proposed method aims to improve target discriminability and detection stability for nighttime monocular RGB inputs without relying on additional sensors, and to provide a methodological reference for future front-end visual perception research in low-altitude intelligent networks.

## 3. Model and Methods

### 3.1. Overall Network Architecture

This study proposes GSC-YOLO, an application-oriented structural optimization framework for low-light monocular RGB pedestrian detection [43]. To address feature degradation, unstable cross-scale fusion, and high-level semantic redundancy in nighttime visible-light scenes, the YOLOv13 baseline [18] is collaboratively redesigned in a task-driven manner. While preserving the original end-to-end detection paradigm [44] and multi-scale prediction mechanism, the proposed framework introduces targeted optimization at three critical stages, namely backbone feature extraction, information filtering during feature fusion, and high-level semantic aggregation, so as to improve detection stability and representational capability in complex low-light environments.

Specifically, GhostNetV3 is adopted as the backbone to enhance multi-scale feature generation under weak-texture conditions with reduced redundancy, thereby alleviating the decline in feature discriminability caused by low-light inputs. During the feature fusion stage, the SSA is introduced to address the inconsistency in nighttime detection whereby deep features provide stronger semantics but insufficient localization details, whereas shallow features retain richer details but are more susceptible to noise contamination [20,45]. Specifically, SDI [46] is first employed to enable cross-scale semantic–detail interaction, after which CBAM performs joint channel–spatial filtering on the fused features to achieve effective information compensation and suppression of redundant responses [47]. Furthermore, to mitigate the problem of dense information distribution and redundant accumulation in high-level semantic branches, C2f_Faster is incorporated into high-semantic layers such as P5 to strengthen semantic aggregation and discriminative representation through a more efficient information flow organization strategy.

**Figure 1:**
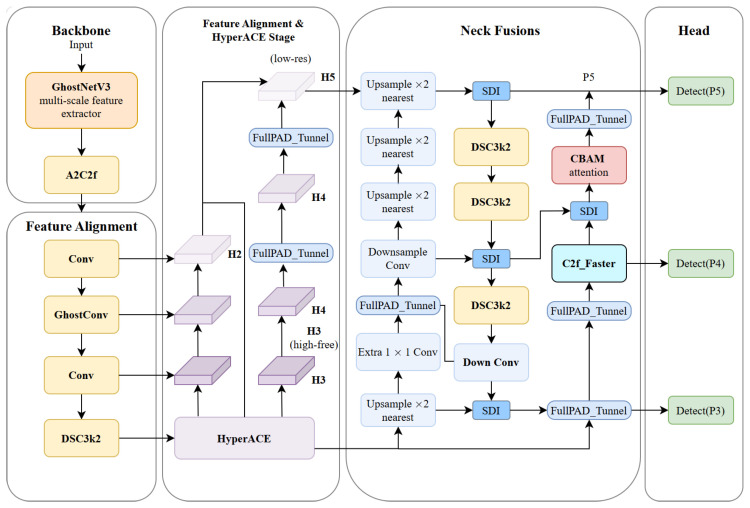
Overall network structure diagram.

Through the above design, the proposed model aims to improve feature representation quality and cross-scale fusion stability in nighttime visible-light scenes without relying on additional sensor inputs. The overall architecture of the improved network is illustrated in Figure 1.

### 3.2. Improved Backbone Network: GhostNetV3

To enhance feature representation under nighttime low-light conditions, GhostNetV3 is adopted as the backbone network in the proposed GSC-YOLO framework. By employing a strategy of primary feature generation followed by inexpensive linear transformations, richer feature maps are produced, thereby alleviating the decline in feature discriminability caused by texture loss in low-light environments.

#### 3.2.1. Ghost Bottleneck Architecture

**Figure 2:**
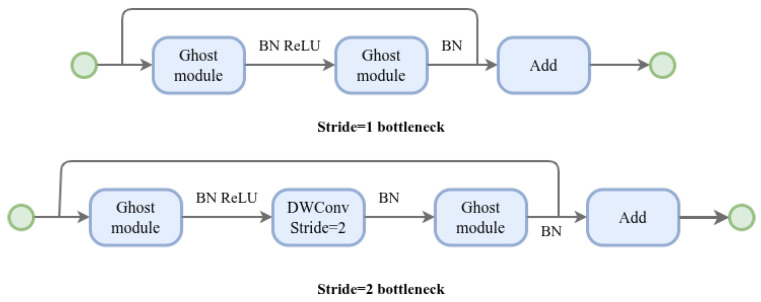
Ghost bottleneck. **Top**: Ghost bottleneck with stride 1. **Bottom**: Ghost bottleneck with stride 2.

Building upon the efficient feature generation mechanism of the Ghost module [48], this study further adopts the Ghost bottleneck (G-bneck) structure designed for lightweight convolutional networks. As illustrated in Figure 2, this structure is conceptually similar to the basic residual block in ResNet [37,39], consisting of multiple convolutional operations and shortcut connections. The main body of the G-bneck consists of two Ghost modules arranged in series. The first Ghost module acts as an expansion layer, increasing the channel dimensionality, where the ratio between the output and input channels is defined as the expansion ratio. The second Ghost module compresses the channel dimension back to match that of the shortcut branch, enabling residual fusion. Finally, the input and output are combined through a shortcut connection to form a residual pathway.

#### 3.2.2. GhostNetV3 Backbone Network

Based on the Ghost bottleneck architecture, GhostNetV3 is adopted as the backbone network of GSC-YOLO. The overall design draws inspiration from the lightweight philosophy of MobileNetV3, while organizing the backbone outputs into a multi-scale feature list tailored for detection tasks, enabling subsequent cross-scale fusion in the neck network and detection head. Specifically, GhostNetV3 performs progressive downsampling across different stages while extracting features with increasingly rich semantic representations. The network ultimately outputs three feature maps corresponding to the commonly used feature pyramid levels in detection tasks—P3, P4, and P5—which represent high-resolution detailed features, medium-scale semantic features, and low-resolution high-level semantic features, respectively. Subsequently, A2C2f is applied to further enhance the highest-level semantic features, making the backbone outputs better suited for the fusion and prediction requirements of the subsequent detection head.

### 3.3. Semantic—Spatial Alignment

In nighttime low-light scenes, deep and shallow features often exhibit a clear mismatch: deep features provide stronger semantic discriminability but lack sufficient localization details, whereas shallow features preserve richer spatial structure while being more susceptible to noise and background interference. When these heterogeneous features are fused only through coarse-grained concatenation, genuinely useful target cues are often difficult to preserve in a stable manner. Therefore, effective alignment between semantic information and spatial details during cross-layer fusion is critical to improving the robustness of low-light pedestrian detection. To address this issue, we propose the Semantic–Spatial Alignment(SSA) module.

#### 3.3.1. Semantic–Detail Interaction Module

**Figure 3:**
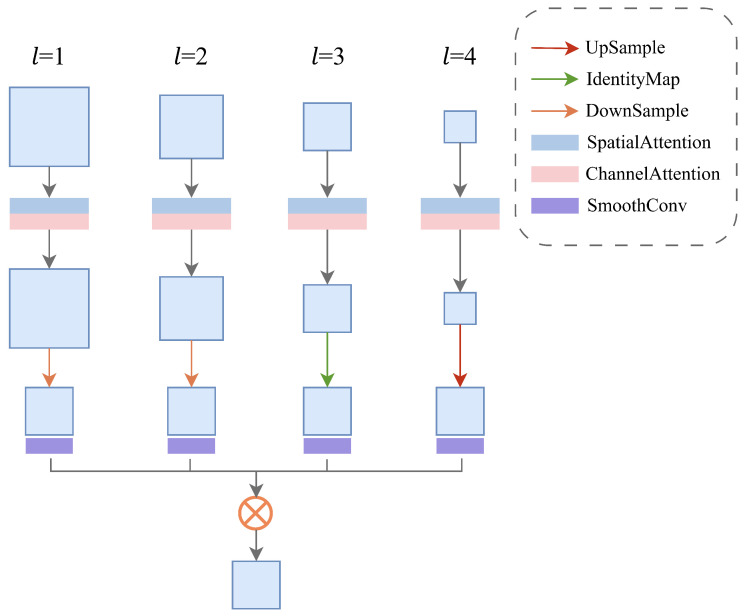
Architecture of the SDI module. SmoothConv denotes a 3×3 convolution for feature smoothing. *H* represents the Hadamard product.

For the multi-level feature maps produced by the encoder, we first apply spatial attention and channel attention to each level, jointly modeling local spatial responses and global channel-wise dependencies. Next, taking the resolution of the i-th feature level $\left(H_{i},W_{i}\right)$ as the target scale, we resize the remaining feature maps to the same spatial size using adaptive average pooling, identity mapping, or bilinear interpolation. A subsequent 3×3 convolution is then applied to smooth the aligned features, mitigating information distortion and aliasing artifacts introduced by resampling. Based on these aligned representations, we perform an element-wise Hadamard product (as shown in Figure 3) over all features associated with the i-th level to enable cross-level interaction and enhancement, yielding output features with both stronger semantic representation and finer detail characterization. The resulting fused features are finally fed into the corresponding level of the decoder for subsequent resolution restoration and the downstream detection task.

#### 3.3.2. Convolutional Block Attention Module

Given an intermediate feature map $F\in R^{C\times H\times W}$ as input, CBAM sequentially derives a 1D channel attention map $M_{c}\in R^{C\times 1\times 1}$ and a 2D spatial attention map $M_{s}\in R^{1\times H\times W}$, The overall attention process can be summarized as follows:

(1) $$F^{\prime }=M_{c}\left(F\right)⊗F,\;F^{\prime \prime }=M_{s}\left(F^{\prime }\right)⊗F^{\prime }$$

where ⊗ denotes element-wise multiplication.

**Figure 4:**
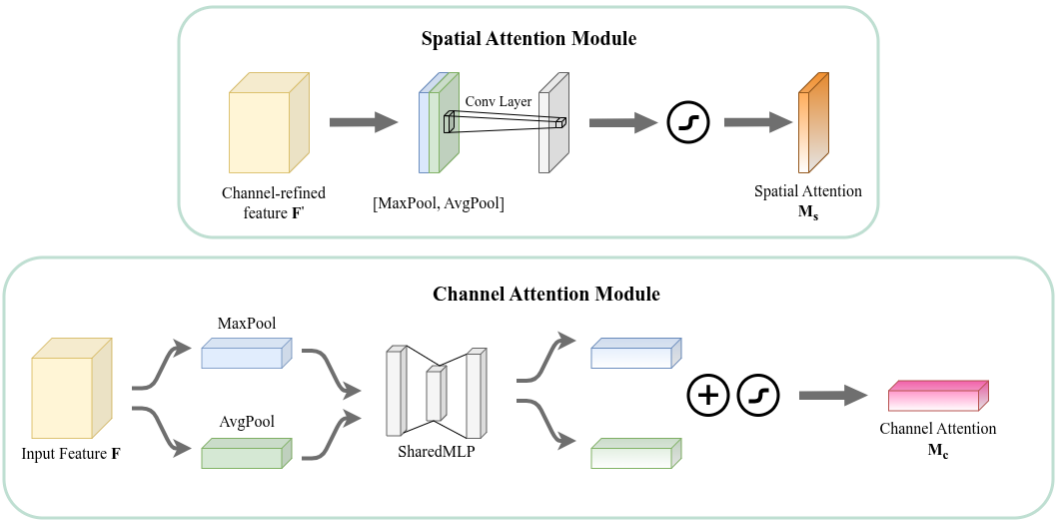
Schematic diagram of the structure of two attention sub modules in CBAM. The channel submodule utilizes both maximum pooling output and average pooling output, and processes them through a shared network; The spatial submodule utilizes two descriptors pooled along the channel axis and sends them to the convolutional layer to generate a spatial attention map.

The computation processes of the two attention maps are illustrated in Figure 4.

#### 3.3.3. Structural Definition and Working Mechanism of SSA

The design rationale of the Semantic–Spatial Alignment module (SSA) is closely associated with the typical failure modes encountered in nighttime low-light detection. In low-light scenes, the challenge lies not only in the susceptibility of shallow features to noise contamination, but also in the fact that weak targets themselves often lack sufficient semantic support. Although shallow features preserve richer spatial details, their discriminative capability remains limited; by contrast, deep features possess stronger semantic representation capacity but are relatively insufficient in localization details. Therefore, before attention-based filtering is applied, the effective information of weak targets should first be supplemented through cross-layer interaction. Based on this consideration, the first stage of SSA employs SDI to perform guided interaction and information injection between deep semantic features and shallow detailed features, thereby sufficiently enhancing weak-target responses during feature fusion. However, although feature representations become richer after SDI-based fusion, redundant activations and certain low-level noise may also be introduced simultaneously. Without further filtering, these irrelevant responses may still interfere with subsequent detection. Accordingly, the second stage of SSA adopts CBAM to perform adaptive recalibration of the fused features along both channel and spatial dimensions, so as to suppress irrelevant background responses and enhance the saliency of target regions, thereby further alleviating noise propagation.

**Figure 5:**
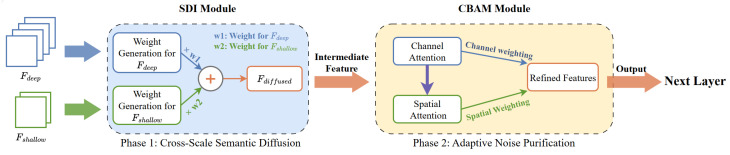
Semantic–Spatial Alignment functional structure diagram.

It should be emphasized that the SDI→CBAM sequence in SSA is intentionally designed, rather than being a simple serial combination of two existing modules. If CBAM-based filtering were applied before SDI-based fusion, the attention mechanism would act before the target semantics had been sufficiently complemented, which might prematurely suppress already weak target responses and consequently weaken the effect of subsequent cross-layer semantic injection. Therefore, the fixed “SDI first, CBAM second” order is more consistent with the processing logic of nighttime low-light detection, namely, to first supplement effective information and then refine the fused responses. The overall architecture of SSA is illustrated in Figure 5. From a structural perspective, the fusion process of SSA is defined as follows:

(2) $$F_{\mathrm{diffused}}=\mathrm{SDI}\left(F_{\mathrm{deep}},F_{\mathrm{shallow}}\right),\;F=\mathrm{CBAM}\left(F_{\mathrm{diffused}}\right)$$

where $F_{\mathrm{deep}}$ and $F_{\mathrm{shallow}}$ denote the features from the deep semantic branch and the shallow detail branch, respectively. $F_{\mathrm{diffused}}$ represents the output after SDI-based interactive fusion, while *F* denotes the refined fusion feature recalibrated by CBAM. SDI is responsible for achieving more comprehensive cross-scale information fusion, whereas CBAM suppresses noise propagation and stabilizes responses in target regions without disrupting the fusion structure. Functionally, the two components complement each other, jointly enhancing the representation capability and stability of fused features in nighttime low-light scenarios.

### 3.4. C2f_Faster Module for High-Level Semantic Aggregation

**Figure 6:**
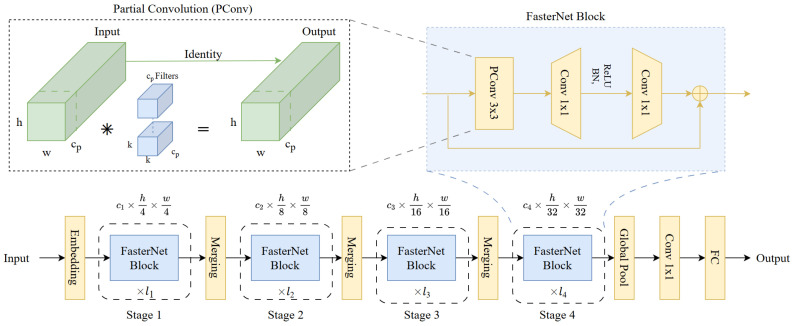
Schematic illustration of the high-level semantic aggregation structure adopted in C2f_Faster. The structure combines PConv and PWConv to optimize high-level semantic information flow through partial-channel spatial mixing and full-channel reorganization while controlling computational overhead.

In this study, C2f_Faster primarily serves an auxiliary role in optimizing high-level semantic information flow and controlling redundant computation. Its purpose is not to independently drive a substantial improvement in detection accuracy, but rather to preserve more effectively the performance gains brought by backbone enhancement and fusion optimization while maintaining overall computational complexity under control. The core idea of C2f_Faster [49] is derived from Partial Convolution (PConv), which performs 3×3 spatial mixing on only a subset of channels while keeping the remaining channels directly connected, as illustrated by PConv in Figure 6.

In our implementation, given a feature map with channel dimension *C*, only C/ndiv channels are subjected to 3×3 convolution, while the remaining channels bypass spatial convolution. This design reduces redundant spatial computation and memory access overhead [50,51]. The computational cost and memory access of PConv can be approximated as follows:

(3) $${\mathrm{FLOPs}}_{\mathrm{PConv}}=h\times w\times k^{2}\times c_{p}^{2},\;{\mathrm{Mem}}_{\mathrm{PConv}}\approx h\times w\times 2c_{p}$$

where $c_{p}$ denotes the number of channels involved in spatial convolution. When $c_{p}=C/4$, the computational overhead of spatial convolution is significantly reduced while still preserving essential local spatial modeling capability.

To compensate for the potentially limited cross-channel interaction introduced by partial-channel convolution, C2f_Faster further incorporates 1×1 channel mixing after spatial mixing, thereby enabling full-channel information reorganization and re-encoding. Given that the P5 layer is characterized by low spatial resolution and strong semantic abstraction, C2f_Faster is primarily deployed at high-semantic levels to optimize the process of high-level semantic aggregation while keeping the overall computational overhead under control.

## 4. Experiment Results

### 4.1. Dataset

To comprehensively evaluate the detection performance and robustness of the proposed model under low-light conditions, complex backgrounds, and multi-scale targets, experiments are conducted on two publicly available datasets: LLVIP [19] and KAIST [20], as shown in Table 1. To ensure a unified evaluation protocol across the two pure RGB benchmark datasets and to enable a fair comparison among all competing models under identical training conditions, the RGB subsets of LLVIP and KAIST were both re-divided into training, validation, and test sets at a ratio of 7:2:1. This design was adopted because the present study focuses on a consistent monocular low-light pedestrian detection setting, rather than directly inheriting heterogeneous protocols originally developed for different multimodal tasks or dataset-specific evaluation objectives. It should be noted that this re-splitting strategy was intended to support internally consistent comparisons and ablation analyses within the scope of this work.

First, the LLVIP (Low-Light Visible–Infrared Pedestrian) dataset is used to establish the primary nighttime detection benchmark and to evaluate the model’s capability for target representation under extremely low-light conditions. The dataset originally contains 15,489 pairs of visible and infrared images. To strictly evaluate nighttime pedestrian detection performance under single-modality visible-light input, only the visible (RGB) images are used, while the infrared modality is excluded. The selected RGB images contain numerous scenarios involving pedestrian occlusion, uneven illumination, and high noise, which effectively reflect the perceptual challenges encountered in real-world nighttime surveillance tasks [52,53].

Furthermore, to evaluate the adaptability of the model in dynamic traffic environments, particularly for detecting pedestrians at different scales [48], the KAIST Multispectral Pedestrian dataset is also introduced. This dataset contains 8995 multispectral images captured in urban road environments, providing abundant nighttime data from vehicle-mounted viewpoints [54]. Factors such as headlight glare, road surface reflections, shadow occlusions, and dynamic backgrounds in this dataset provide effective support for evaluating the model’s noise robustness and multi-scale perception capability in complex nighttime environments [55].

In this study, LLVIP was primarily employed to validate the detector’s performance under extremely low-light nighttime conditions, whereas the RGB subset of KAIST was introduced as a complementary benchmark to evaluate robustness in dynamic urban nighttime scenes characterized by glare, reflections, scale variations, and complex backgrounds.

### 4.2. Experimental Environment and Setting

**Figure 7:**
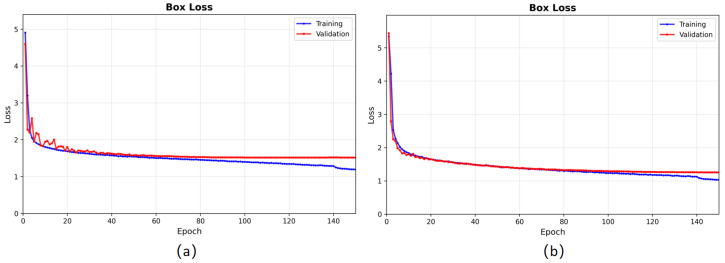
Loss curves during training. Blue: training; red: validation. Both losses converge smoothly, indicating effective model training. (**a**) Loss trend observed on the LLVIP dataset. (**b**) Loss trend observed on the KAIST dataset.

All experiments were conducted on a computing platform equipped with both CPU and GPU. The key training hyperparameters are summarized in Table 2, and the detailed hardware and software configurations are reported in Table 3. As illustrated in Figure 7, the model had converged by 150 training epochs, and early stopping was enabled with a patience of 30 to avoid resource waste when performance no longer improved [56].

### 4.3. Evaluation Metrics

To objectively and comprehensively evaluate the performance of the GSC-YOLO model in nighttime pedestrian detection, this study adopts a series of standardized metrics, including precision (P), recall (R), mean average precision (mAP), and the F1 score. Precision reflects the model’s ability to control false positives, whereas recall measures the completeness of target detection. In safety-critical tasks such as nighttime security inspection and patrol, recall is particularly important because missed detections may lead to greater practical risks. The mathematical definitions of precision and recall are as follows:

(4) $$P=\frac{TP}{TP+FP}$$

(5) $$R=\frac{TP}{TP+FN}$$

where TP denotes true positives, FP denotes false positives, and FN denotes false negatives.

Mean average precision (mAP) is a core indicator for evaluating detection performance. In this study, both mAP@0.5 and mAP@0.5:0.95 are reported. Specifically, mAP@0.5 represents the average precision at an IoU threshold of 0.5, whereas mAP@0.5:0.95 denotes the mean of average precision values computed over IoU thresholds ranging from 0.5 to 0.95 with a step size of 0.05. Compared with a single IoU threshold, the latter imposes stricter requirements on bounding-box localization accuracy and therefore provides a more comprehensive reflection of detection quality. Its calculation is given as follows:

(6) $$\mathrm{mAP}=\frac{1}{M}\sum_{m=1}^{M}AP_{m}$$

where *M* is the total number of classes (only the pedestrian class in this study), and $AP_{m}$ is the average precision of the *m*-th class.

To comprehensively assess the balance between precision and recall, the F1 score is also adopted as an auxiliary metric in this study. It is calculated as follows:

(7) $$F1=\frac{2\times P\times R}{P+R}$$

In addition to detection accuracy, this study also evaluates model complexity and real-time performance. Among these, FLOPs are used to measure the number of floating-point operations required for a single forward inference pass, thereby indirectly reflecting the model’s computational demand on hardware [57]. For convolution operations, the calculation can be expressed as follows:

(8) $$\mathrm{FLOPs}=2\times H\times W\times C_{\mathrm{in}}\times K^{2}\times C_{\mathrm{out}}$$

where $C_{\mathrm{in}}$ denotes the number of channels in the input tensor of the convolutional layer, $C_{\mathrm{out}}$ denotes the number of channels in the output tensor, and *K* denotes the kernel size.

In addition, frames per second (FPS) is used to evaluate the real-time inference capability of the model and is defined as follows:

(9) $$\mathrm{FPS}=\frac{1}{t_{\mathrm{pre}}+t_{\inf}+t_{\mathrm{post}}}$$

where $t_{\mathrm{pre}}$ represents the preprocessing time, $t_{\inf}$ denotes the inference time, and $t_{\mathrm{post}}$ denotes the post-processing time.

### 4.4. Comparative Experimental Analysis

**Figure 8:**
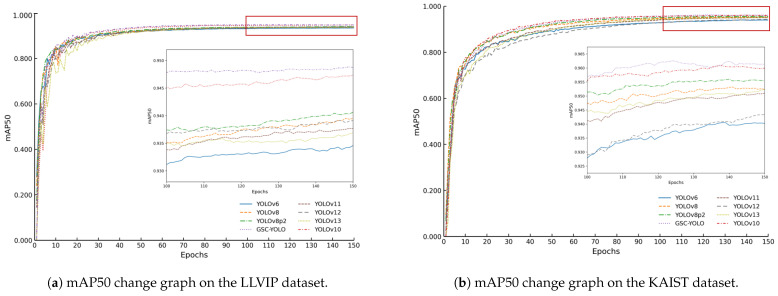
Curves of model evaluation metrics. (**a**) The mean Average Precision at 50% IoU threshold (mAP50) metric change graph of GSC-YOLO under the LLVIP dataset. (**b**) The mean Average Precision at 50% IoU threshold (mAP50) metric change graph of GSC-YOLO under the KAIST dataset.

This section first compares the proposed GSC-YOLO with mainstream YOLO-series models on two public benchmarks, LLVIP and KAIST. Subsequently, ablation experiments are conducted to analyze the individual contributions and collaborative effects of three core modules: GhostNetV3, SSA, and C2f_Faster. The experimental data are drawn from public nighttime pedestrian detection benchmarks and are mainly used to validate the effectiveness of the proposed model in weak-target perception, noise suppression, and real-time detection under low-light conditions [58]. It should also be noted that direct experimental comparison with RGB-T or multispectral detectors is not included in this study, because the present work is conducted strictly under a monocular RGB setting and focuses on detection performance as well as the accuracy–complexity trade-off without introducing additional infrared hardware or cross-modal calibration overhead. Therefore, the conclusions of this paper should be interpreted primarily as evidence of the effectiveness of the proposed method within the monocular RGB setting, rather than as a claim of superiority over multimodal approaches. The model evaluation metric curve is shown in Figure 8.

#### 4.4.1. Performance Analysis on the LLVIP Dataset

On the LLVIP low-light dataset, GSC-YOLO demonstrates strong overall detection performance. As shown in Table 4, the proposed method achieves an mAP@0.5:0.95 of 57.70%, improving upon the baseline YOLOv13 (54.45%) by 3.25 percentage points and surpassing the best competing model, YOLOv10 (56.92%). In terms of recall, GSC-YOLO achieves 89.93%, exceeding all comparison models, with YOLOv10 ranking second at 88.99%. Meanwhile, GSC-YOLO attains a precision of 93.48% and an F1-score of 91.67%, indicating that the model improves detection capability while maintaining a good balance between precision and recall. In terms of efficiency, GSC-YOLO requires 13.38 GFLOPs, higher than the baseline YOLOv13 (3.06 GFLOPs), and achieves an inference speed of 45.35 FPS compared with the baseline’s 102.16 FPS. Although the introduced structural enhancement modules increase computational cost, the model still maintains real-time detection capability [59]. On the RTX 4090D (24 GB) platform used in this study, GSC-YOLO achieves improved accuracy while retaining satisfactory online inference performance.

#### 4.4.2. Performance Analysis on the KAIST Dataset

Compared with LLVIP, the KAIST dataset contains more nighttime vehicle-mounted scene images, where factors such as headlight glare, road reflections, and dynamic shadows more easily lead to false positives and missed detections. As shown in Table 5, GSC-YOLO also achieves the best overall performance on this dataset. Specifically, the proposed method attains an mAP@0.5:0.95 of 66.61%, representing an improvement of 2.50 percentage points over the baseline YOLOv13 (64.11%) and outperforming all other comparison methods.

In terms of precision, GSC-YOLO achieves 0.9453, the highest among all comparison models, indicating that the proposed method effectively suppresses background interference and false positives in complex nighttime environments. Meanwhile, the recall reaches 90.49%, exceeding that of YOLOv13 (88.79%) and YOLOv10 (90.24%), demonstrating that the model maintains strong target coverage in dynamic scenes. Overall, GSC-YOLO achieves an F1-score of 92.47% on KAIST, further confirming a well-balanced trade-off between precision and recall.

In terms of efficiency, GSC-YOLO requires 13.38 GFLOPs and achieves an inference speed of 47.82 FPS. Although the speed is lower than that of the lightweight baseline model, it still satisfies real-time processing requirements.

Considering the experimental results on both the LLVIP and KAIST datasets, GSC-YOLO demonstrates stable detection performance across different types of low-light scenarios. Whether in static low-light surveillance environments (LLVIP) or dynamic urban driving scenes (KAIST), the proposed method achieves a reasonable balance between detection accuracy and real-time performance. Because our experimental setting is restricted to monocular RGB input, the comparative results reported here are intended to validate the effectiveness of the proposed method within a pure RGB low-light detection setting. Accordingly, these results should not be interpreted as demonstrating superiority over multimodal systems, which, although benefiting from additional sensing modalities, also incur higher hardware costs and cross-modal alignment overhead.

### 4.5. Ablation Experimental

To quantify the contribution of each proposed module to the model performance, systematic ablation experiments were conducted on the LLVIP and KAIST datasets. Using YOLOv13 as the baseline, the GhostNetV3 backbone, SSA module, and C2f_Faster aggregation module were gradually introduced. The corresponding experimental results are presented in Table 6 and Table 7.

On the LLVIP dataset, adopting GhostNetV3 as the backbone (Table 6, Model 2) yields the most significant performance improvement. The mAP@0.5:0.95 increases from the baseline value of 54.45% to 57.31%, representing a gain of 2.86 percentage points, while recall improves from 88.35% to 90.09%. This result indicates that stronger backbone representational capacity is the primary source of the overall performance improvement. Although the GFLOPs increased from 3.06 G to 10.89 G, GhostNetV3 substantially enhanced multi-scale feature representation under weak-texture conditions in low-light nighttime scenes, thereby providing a more effective representational foundation for subsequent detection.

By contrast, when SSA was introduced alone (Table 6, Model 3), the mAP@0.5:0.95 on LLVIP increased to 55.61%, representing an improvement of 1.16 percentage points over the baseline. This result suggests that, although the standalone gain of SSA is smaller than that achieved by backbone replacement, its value lies primarily in the targeted optimization of semantic compensation for weak targets and noise suppression during cross-layer fusion, rather than in the additional benefit brought by simply stacking an attention mechanism. On the other hand, when C2f_Faster was used alone (Table 6, Model 4), the mAP@0.5:0.95 increased only marginally from 54.45% to 54.55%, while the recall decreased from 88.35% to 87.72%. This indicates that the role of C2f_Faster is more closely related to the organization of high-level semantic information flow and efficiency control, rather than to the independent enhancement of low-level feature representation.

From the results of module combinations, after GhostNetV3 was combined with SSA (Table 6, Model 5), the mAP@0.5:0.95 further increased to 57.59%, indicating that SSA can continue to improve cross-layer fusion quality on top of enhanced backbone representation. When all three modules were enabled simultaneously (Table 6, Model 8), the model achieved the best performance on LLVIP, with the mAP@0.5:0.95 reaching 57.70% and the F1-score reaching 91.67%. Notably, compared with Model 5, the introduction of C2f_Faster reduced the GFLOPs from 15.25 G to 13.38 G while still further improving accuracy. This suggests that, although C2f_Faster is not the primary source of accuracy gains, it can preserve more effectively the performance benefits introduced by backbone enhancement and fusion optimization while controlling model complexity.

A consistent trend was also observed on the KAIST dataset. The full model (Table 7, Model 8) achieved the highest mAP@0.5:0.95 of 66.61% among all configurations, with a complexity of 13.38 G, indicating that the proposed structural optimization strategy exhibits good adaptability across different nighttime scenarios.

Overall, the ablation results show that GhostNetV3 delivers the most substantial standalone performance improvement, indicating that stronger backbone representational capacity is the principal source of the overall gain. By comparison, the standalone gain of SSA is relatively modest, but its value mainly lies in the targeted optimization of semantic compensation for weak targets and noise suppression during cross-layer fusion. C2f_Faster, in turn, contributes primarily to the organization of high-level semantic information flow and efficiency control, rather than to the independent enhancement of low-level representational capacity.

### 4.6. Discussion on the Trade-Off Between Efficiency and Accuracy

The experimental results indicate that, although GSC-YOLO significantly improves detection accuracy and recall, these gains are inevitably accompanied by additional computational overhead and a reduction in inference speed. Nevertheless, such a trade-off is acceptable from an engineering perspective. First, on the RTX 4090D (24 GB) platform used in this study, the proposed model achieved 45.35 FPS on LLVIP and 47.82 FPS on KAIST, respectively, indicating that it still provides favorable online processing performance in a desktop-class GPU environment. Second, in low-light monocular RGB scenarios, a moderate increase in computational cost can be exchanged for higher recall and more stable target localization, which is of practical significance for safety-critical applications such as nighttime security surveillance and front-end visual perception in low-altitude intelligent networks. Therefore, the purpose of discussing the accuracy–efficiency relationship in this work is not to claim that the model already satisfies the deployment requirements of all resource-constrained platforms, but rather to demonstrate that the proposed structural optimization achieves a comparatively reasonable balance among accuracy, recall, and complexity under the current experimental conditions.

### 4.7. Visualization Analysis

To more intuitively validate the detection performance of GSC-YOLO in scenarios with complex illumination and weakly visible targets, several representative examples were selected from the LLVIP (RGB) and KAIST (RGB) datasets and visually compared side by side with the baseline model, YOLOv13. The detection results of the baseline model are marked with red bounding boxes, whereas those of the proposed method are indicated with green bounding boxes.

**Figure 9:**
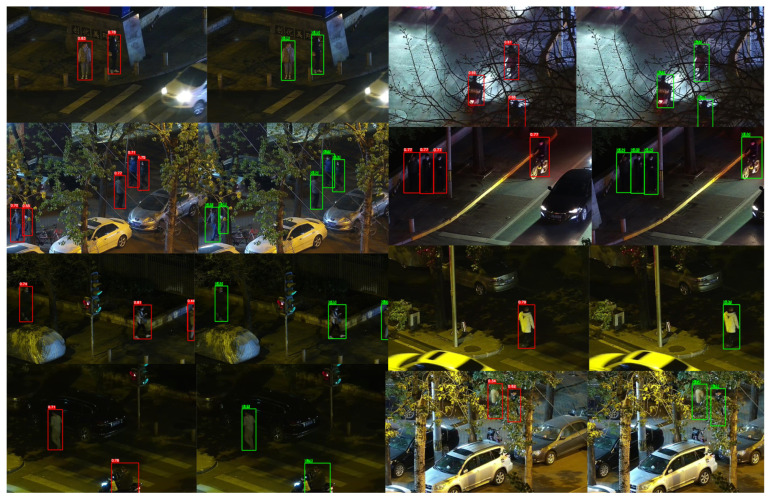
Side-by-side qualitative comparison on the LLVIP dataset. Red boxes denote YOLOv13 detections, and green boxes denote GSC-YOLO detections.

As illustrated in Figure 9, nighttime scenes in the LLVIP dataset are typically affected by uneven illumination, local strong-light interference, large dark regions, and occlusion from complex backgrounds, which weaken the texture details of pedestrian targets and blur their boundaries, thereby posing substantial challenges to the detection task. Compared with the baseline model, GSC-YOLO exhibits more stable responses when detecting weak targets in dark regions, targets in tree-occluded areas, and targets located near vehicles or within cluttered backgrounds. Moreover, in scenes where partial occlusion coexists with small-scale targets, GSC-YOLO achieves more stable detections, suggesting stronger weak-target perception capability and greater resistance to interference in complex nighttime backgrounds. Overall, compared with the baseline model, GSC-YOLO demonstrates more robust detection performance for targets in dark regions, weakly salient targets, and targets embedded in complex backgrounds under nighttime low-light conditions in LLVIP.

**Figure 10:**
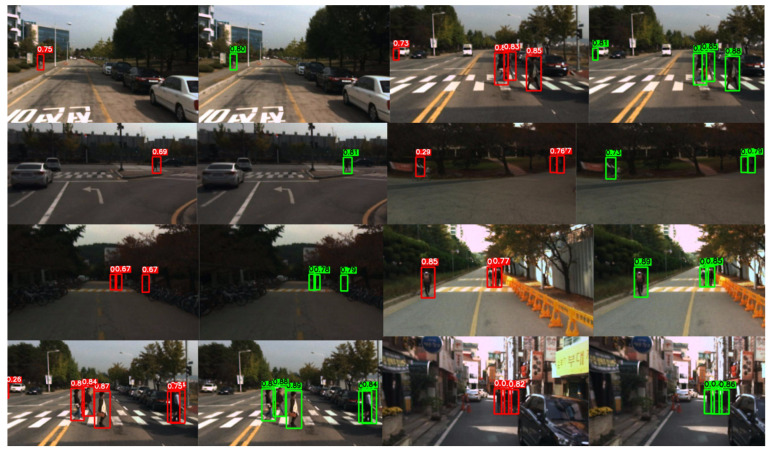
Side-by-side qualitative comparison on the KAIST dataset. Red boxes denote YOLOv13 detections, and green boxes denote GSC-YOLO detections.

Further observations from the KAIST dataset examples in Figure 10 show that GSC-YOLO provides better detection responses to distant small targets, targets near scene boundaries, and pedestrians embedded in low-contrast backgrounds under typical street-scene conditions. Compared with the baseline YOLOv13, the proposed method is able to detect weak and small pedestrians located in the far road region more consistently in several examples, while maintaining better response completeness when multiple targets appear simultaneously. For street-scene examples captured at dusk, during low-light daytime conditions, and at night with background shadows or local illumination variations, GSC-YOLO also exhibits more stable detection results, indicating that the proposed method offers certain advantages in multi-scale target representation and adaptation to complex visible-light conditions. In particular, for targets that are distant, weakly salient, or located near image boundaries, the proposed method is less prone than the baseline to insufficient responses or missed detections.

### 4.8. Error Case Analysis

**Figure 11:**
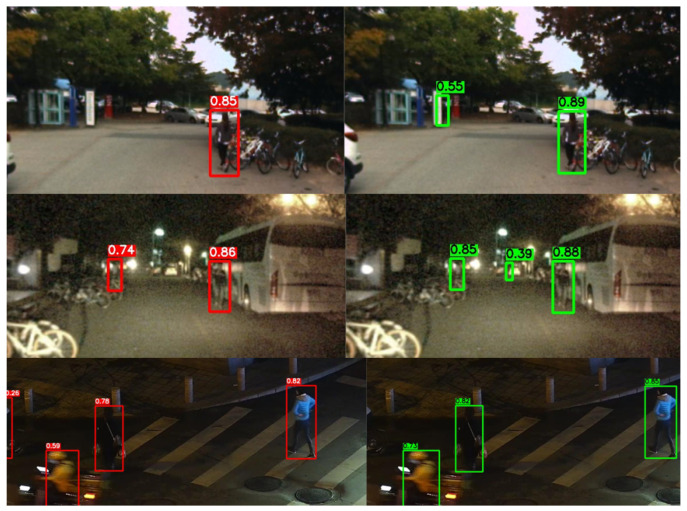
Representative failure cases. The first two groups show false positives of GSC-YOLO, while the third group shows a missed detection relative to the baseline.

Although GSC-YOLO achieves favorable performance on both datasets, it still exhibits certain limitations in extremely complex scenarios, as illustrated in Figure 11. In the first group of examples, GSC-YOLO produced an additional bounding box in a distant background region where no actual pedestrian was present, resulting in a false positive. This suggests that under low-contrast conditions, the model may still be affected by vertical background structures and weak-texture regions. In the second group, the proposed method also generated an additional false positive under a strongly noisy nighttime background, indicating that under extremely low-light and blurred-background conditions, the enhancement of weak responses may also lead to a certain degree of spurious target activation. In the third group, GSC-YOLO detected one fewer target than the baseline, resulting in a false negative. This indicates that under conditions involving multiple coexisting targets, local motion interference, or blurred boundaries, the model may still suffer from insufficient target coverage. Overall, these failure cases show that the proposed method still faces the coexistence of false positives and false negatives in complex nighttime scenes. Future work may further improve robustness through enhanced background suppression, more discriminative small-target modeling, and better dynamic target representation.

## 5. Conclusions

This study proposes and validates GSC-YOLO, a structural optimization model designed for pedestrian detection in low-light monocular RGB scenarios. To address challenges associated with weak texture, strong noise, and insufficient efficiency in multi-scale feature fusion, the model introduces GhostNetV3 as the backbone to enhance feature representation, designs the Semantic–Spatial Alignment module (SSA), and adopts C2f_Faster to improve the efficiency of high-level feature aggregation.

Experimental results on the LLVIP and KAIST datasets demonstrate that GSC-YOLO achieves strong performance in low-light pedestrian detection. Compared with the baseline model, GSC-YOLO improves mAP@0.5:0.95 by 3.25% and 2.50%, respectively, and achieves the highest recall on both datasets, helping reduce the risk of missed detections in nighttime safety-related scenarios. Although the computational cost increases to 13.38 GFLOPs, the model still achieves 45.35 FPS and 47.82 FPS on the experimental platform, indicating that it maintains favorable real-time performance while improving detection accuracy and achieves a comparatively reasonable balance between accuracy and efficiency.

Qualitative results further show that the proposed method can detect more valid targets under challenging conditions such as severe occlusion, headlight glare, and low-contrast backgrounds, although false positives and false negatives still occur in scenarios involving long-distance targets, strong noise, and multi-target interference. Direct experimental comparison with representative RGB-T or multispectral detection methods has not been included in this study; therefore, the relevant conclusions should be interpreted primarily within the setting of monocular RGB low-light pedestrian detection. Future work will further improve the proposed method through enhanced complex-background suppression, small-target enhancement, and dynamic target modeling. Although this study does not address the design of the control and communication layers, nor does it provide systematic validation on an airborne platform, robust low-light visual perception remains an important component of front-end environmental sensing in the functional chain of low-altitude intelligent networks. The structural optimization method proposed in this study for low-light visible-light pedestrian detection may serve as a methodological reference for future front-end visual sensing research in low-altitude inspection and nighttime risk perception scenarios.

## Figures and Tables

**Table 1:** LLVIP and KAIST dataset description.

Dataset	Source	Training Set	Validation Set	Test Set
LLVIP	Beijing University of Posts and Telecommunications and Xidian University [19]	10,841	3098	1550
KAIST	Korea Advanced Institute of Science and Technology [20]	6296	1799	900

**Table 2:** Training hyperparameter settings.

Parameter	Value
Input Size	640×640
Optimizer	SGD
Momentum	0.937
Batch Size	16
Weight Decay	0.0005
Workers	8
Close Mosaic	10
Pretrained	False

**Table 3:** Hardware and software environment configuration.

Component	Specification/Version
CPU	16 vCPU Intel(R) Xeon(R) Platinum 8481C
GPU	RTX 4090D (24 GB) × 1
Memory	80 GB
OS	Linux
Deep Learning Framework	PyTorch 2.5
CUDA Version	12.4
Python Version	3.10.16

**Table 4:** Performance comparison of different models on the LLVIP dataset.

Dataset	Model	Precision	Recall	mAP@0.5	mAP@0.5:0.95	F1	GFLOPs	FPS
LLVIP	YOLOv6	93.47%	88.06%	93.44%	55.01%	90.68%	5.89	302.35
	YOLOv8	93.46%	88.24%	93.81%	55.51%	90.78%	4.04	286.59
	YOLOv8p2	93.04%	89.11%	93.94%	56.02%	91.03%	6.09	252.64
	YOLOv10	93.49%	88.99%	94.55%	56.92%	91.18%	12.23	141.69
	YOLOv11	92.93%	88.46%	93.82%	55.51%	90.64%	3.16	217.33
	YOLOv12	93.11%	88.60%	93.88%	55.03%	90.80%	2.91	153.39
	YOLOv13	92.65%	88.35%	93.57%	54.45%	90.45%	3.06	102.16
	Ours	93.48%	89.93%	94.78%	57.70%	91.67%	13.38	45.35

**Table 5:** Performance comparison of different models on the KAIST dataset.

Dataset	Model	Precision	Recall	mAP@0.5	mAP@0.5:0.95	F1	GFLOPs	FPS
KAIST	YOLOv6	92.53%	86.49%	94.03%	62.20%	89.41%	5.89	312.65
	YOLOv8	93.10%	89.41%	95.23%	64.16%	91.22%	4.04	289.07
	YOLOv8p2	92.22%	89.09%	95.43%	64.00%	90.63%	6.09	242.80
	YOLOv10	93.12%	90.24%	95.66%	65.10%	91.66%	12.23	189.15
	YOLOv11	93.33%	88.49%	95.08%	63.93%	90.85%	3.16	229.26
	YOLOv12	92.60%	87.30%	94.34%	62.16%	89.87%	2.91	177.29
	YOLOv13	93.23%	88.79%	95.23%	64.11%	90.96%	3.06	102.38
	Ours	94.53%	90.49%	96.00%	66.61%	92.47%	13.38	47.82

**Table 6:** Ablation results on the LLVIP dataset.

Dataset	Model	GhostNet	SDI+CBAM	C2f_Faster	Precision	Recall	mAP@0.5	mAP@0.5:0.95	F1	GFLOPs	FPS
LLVIP	1				92.65%	88.35%	93.67%	54.45%	90.45%	3.06	102.16
	2	✓			93.20%	90.09%	94.70%	57.31%	91.62%	10.89	49.31
	3		✓		93.25%	88.58%	94.03%	55.61%	90.86%	6.59	95.83
	4			✓	92.80%	87.72%	93.66%	54.55%	90.19%	3.03	111.27
	5	✓	✓		93.56%	89.95%	94.87%	57.59%	91.72%	15.25	47.06
	6		✓	✓	93.48%	89.93%	94.78%	57.56%	91.67%	14.38	43.53
	7	✓		✓	93.71%	89.92%	94.67%	57.27%	91.78%	10.86	48.14
	8	✓	✓	✓	93.48%	89.93%	94.78%	57.70%	91.67%	13.38	45.35

**Table 7:** Ablation results on the KAIST dataset.

Dataset	Model	GhostNet	SDI+CBAM	C2f_Faster	Precision	Recall	mAP@0.5	mAP@0.5:0.95	F1	GFLOPs	FPS
KAIST	1				93.23%	88.79%	95.23%	64.11%	90.96%	3.06	102.38
	2	✓			93.99%	90.60%	95.98%	66.00%	92.26%	10.89	48.88
	3		✓		94.08%	88.36%	95.34%	64.58%	91.13%	6.59	74.61
	4			✓	92.66%	89.31%	95.32%	64.54%	90.95%	3.03	81.72
	5	✓	✓		94.73%	91.03%	96.14%	65.58%	92.84%	12.25	42.76
	6		✓	✓	92.74%	89.52%	95.34%	65.32%	91.10%	6.56	101.42
	7	✓		✓	93.89%	90.69%	95.88%	65.78%	92.26%	10.86	44.51
	8	✓	✓	✓	94.53%	90.49%	96.00%	66.61%	92.47%	13.38	47.82

## Data Availability

The data presented in this study are available upon request from the corresponding author.
